# Supplementary material for: Vesicular Stomatitis Virus Elicits Early Transcriptome Response in Culicoides sonorensis Cells
Source: Viruses. 2023 Oct 18;15(10):2108. doi: 10.3390/v15102108 (PMC10612082; doi:10.3390/v15102108)
Supplement: Supplementary file 1 [file viruses-15-02108-s001.zip › Supplemental Figure S1.pdf]

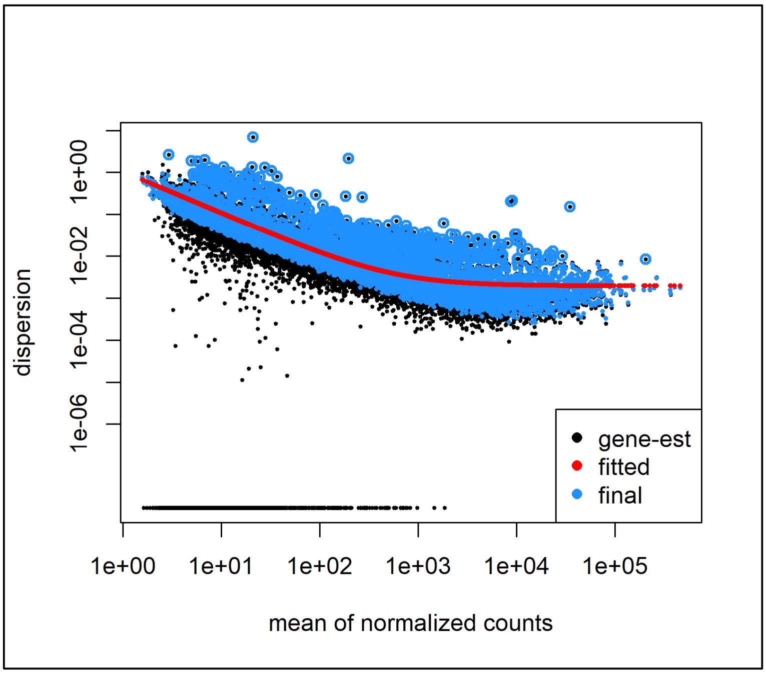

Figure S1. Dispersion plot informs appropriate GLM modeling of variance in differential expression statistical testing. The dispersion plot shows the relationship between dispersion and the average expression level of genes in the differential expression analysis. Dispersion represents the variance of each gene after accounting for the variability expected from the mean expression. Genes with higher dispersion (variance) appear above the fitted curve. Gene-est: genes with an associated mean expression level and maximum likelihood estimation of the dispersion. Fitted: distribution of reasonable estimates of dispersion. Final: genes that follow the likelihood estimates of the dispersion model. The plot was generated by DESeq2 in R.
