# Supplementary material for: Vesicular Stomatitis Virus Elicits Early Transcriptome Response in Culicoides sonorensis Cells
Source: Viruses. 2023 Oct 18;15(10):2108. doi: 10.3390/v15102108 (PMC10612082; doi:10.3390/v15102108)
Supplement: Supplementary file 1 [file viruses-15-02108-s001.zip › Supplemental Figure S2.pdf]

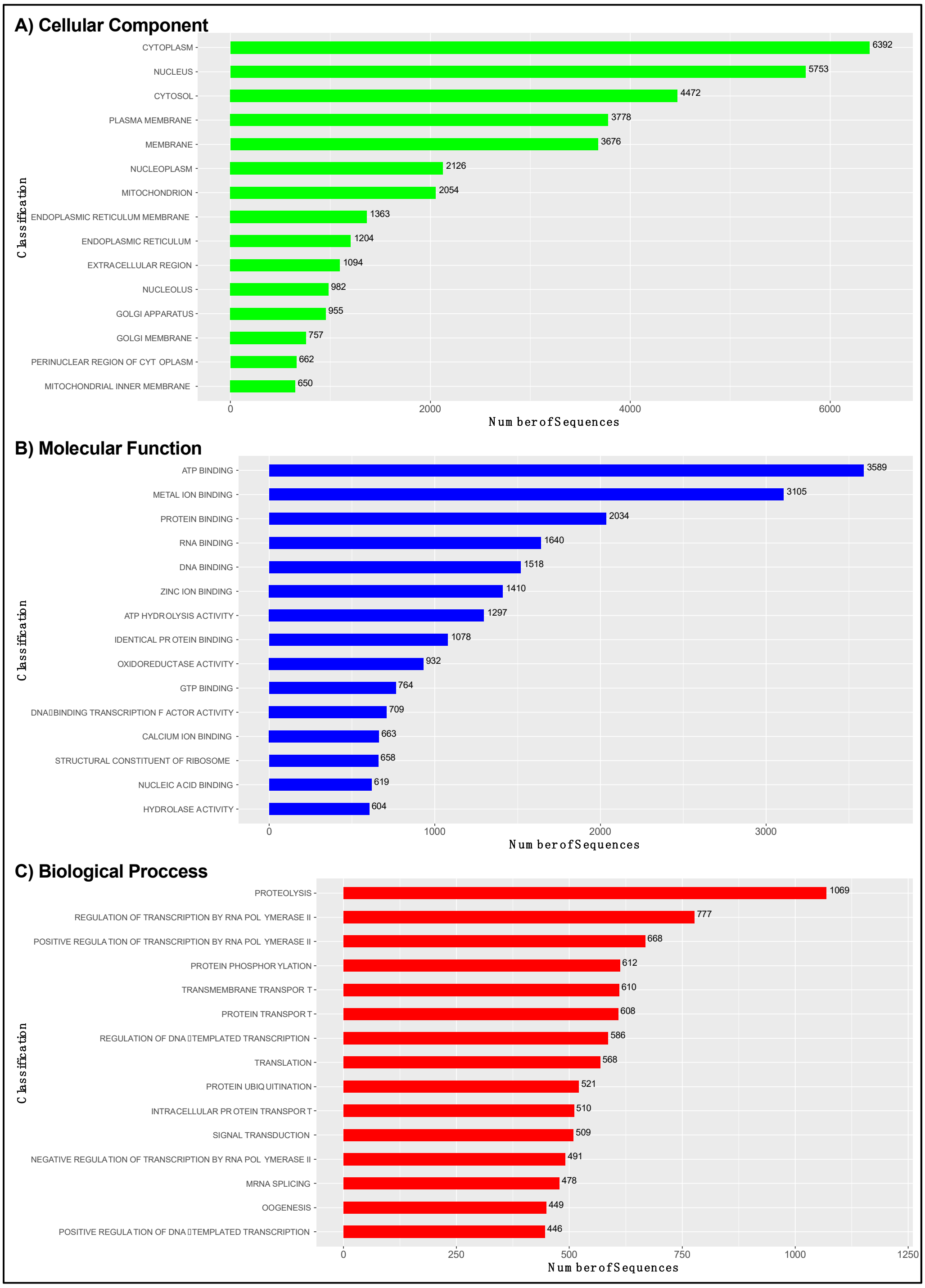


Figure S2. Gene ontology annotations from entire W8 transcriptome A) cellular components, B) Molecular function, C) Biological processes.
