# Supplementary material for: Vesicular Stomatitis Virus Elicits Early Transcriptome Response in Culicoides sonorensis Cells
Source: Viruses. 2023 Oct 18;15(10):2108. doi: 10.3390/v15102108 (PMC10612082; doi:10.3390/v15102108)
Supplement: Supplementary file 1 [file viruses-15-02108-s001.zip › Supplemental Figure S3.pdf]

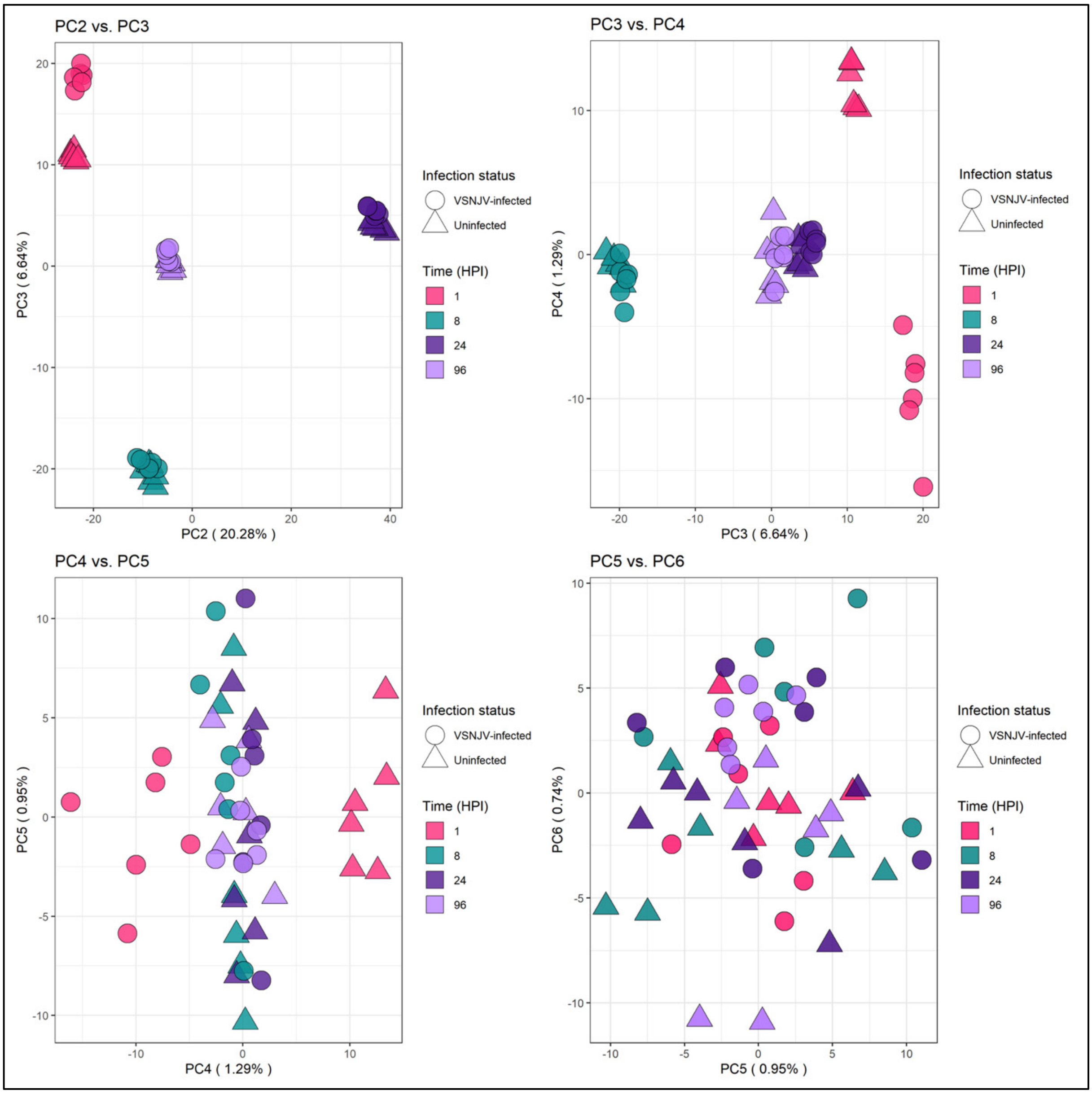

Figure S3. Principal component analyses for transcriptomes from VSNJV-infected and mock infected W8 cells at 4 time points post-infection. See Figure 2 for PC1 vs PC2 and PC1 vs PC3.
